# A case report of primary myxoid liposarcoma mimicking as a parotid cystic lesion

**DOI:** 10.1097/MD.0000000000028473

**Published:** 2022-01-14

**Authors:** Yong Tae Hong, Yunna Yang, Hyunjun Lee

**Affiliations:** Department of Otolaryngology-HNS1, Research Institute for Clinical Medicine of Jeonbuk National University- Biomedical Research Institute of Jeonbuk National University Hospital, Chonbuk, Korea.

**Keywords:** liposarcoma, parotid gland

## Abstract

**Rationale:**

Myxoid liposarcoma (MLS) is an extremely rare tumor of the salivary gland and it arises from undifferentiated pluripotent mesenchymal cells. We report a rare case of a primary MLS in the parotid gland.

**Patient concerns:**

The patient was a 49-year-old female who presented with a hard fixed mass in the left parotid region.

**Diagnosis:**

On computed tomography and MR images, this tumor has a low attenuation center with a thick enhancing wall and ill-defined margins. The absence of high-signal-intensity foci on T1-weighted images makes a MLS indistinguishable from most other soft-tissue masses. Pathologically, the tumor was diagnosed as MLS.

**Interventions:**

The patient received total parotidectomy with facial nerve preservation and selective neck dissection.

**Outcomes:**

Immediate facial nerve function was House Brackmann Grade III and recovered within 3 months after the surgery. Follow-up period is 57 months and there was no recurrence until now.

**Lessons:**

In this report, we report a rare case of primary MLS mimicking a cystic lesion of the parotid gland.

## 1 Introduction

Liposarcoma is generally asymptomatic, and it can occur anywhere in the body and it usually arises in the deep soft tissue of the lower extremity, particularly the thigh.^[1–3]^ Other locations include the chest wall, axilla, shoulder, inguinal region, buttock, and the neck. Primary liposarcoma of the salivary gland is an extremely rare malignancy, and it mostly occurs in the parotid gland. The other major salivary glands (submandibular and sublingual glands) are very rarely involved.^[4]^ When the tumor involves a major salivary gland, it present as a painless mass in the gland. When the minor salivary glands are involved, the liposarcomas may occur in the tongue, buccal cavity, palate, and other mouth regions.^[5]^ The cause of salivary gland liposarcoma is suspected to be a genetic abnormality and no definitive risk factors for liposarcoma development have been observed.^[6]^

The signs and symptoms of liposarcoma of the salivary gland include a slow-growing mass in the parotid area and pain while eating. Appropriate diagnosis and identification of the subtype of liposarcoma are essential for proper management of the subtypes.^[6]^ A biopsy of the tumor is necessary for making an exact diagnosis to start treatment. They are of 4 different types; well-differentiated liposarcoma, dedifferentiated liposarcoma, myxoid liposarcoma (MLS), and pleomorphic liposarcoma. MLS accounts for approximately one third of all liposarcomas and has a predilection for a younger age group, with a peak incidence during the fifth decade of life.^[7]^ Genetic analysis for detecting any chromosomal anomalies can be used for making the initial diagnosis. These results provide supportive evidence for molecular testing when we encounter this differential diagnosis.

The following 4 basic criteria for the diagnosis of primary MLS of the salivary gland are proposed: no sarcoma elsewhere, exclusion of metastasis to the gland from malignancies at other sites, exclusion of a carcinosarcoma, and pathological consistence with origin within the gland.^[8]^ Interestingly, computed tomography (CT) or magnetic resonance imaging (MRI) may not show the typical features of a lipomatous tumor because the fat content of liposarcoma is often less than 10% to 25%.^[6]^ Therefore, it may be difficult to make the correct diagnosis of some MLSs with radiology due to the lack of fat signal intensity. The abnormal findings in magnetic resonance (MR) images also depend on the amount of myxoid material, the degree of cellularity and vascularity, and the presence of necrosis. Thus, some MLSs appear to be cystic on nonenhanced MRI, although they enhance like other solid masses on contrast material–enhanced MRI.^[9–11]^

The clinical prognosis is strongly correlated with the histologic features, a more aggressive course being reported for tumors with a rich round cell component.^[4]^ Metastases to lung, bone, and soft tissue have been reported in the literature. A combined treatment with surgery, chemotherapy, and radiation therapy could be applied for treating MLS of the salivary gland. Overall prognosis is reported to be relatively good after prompt diagnosis and adequate treatment. We report a case of primary MLS of the parotid gland mimicking a cystic parotid lesion with a review of the literature. Informed consent was received from the patients about the publication.

## 2 Case report

**Figure 1:**
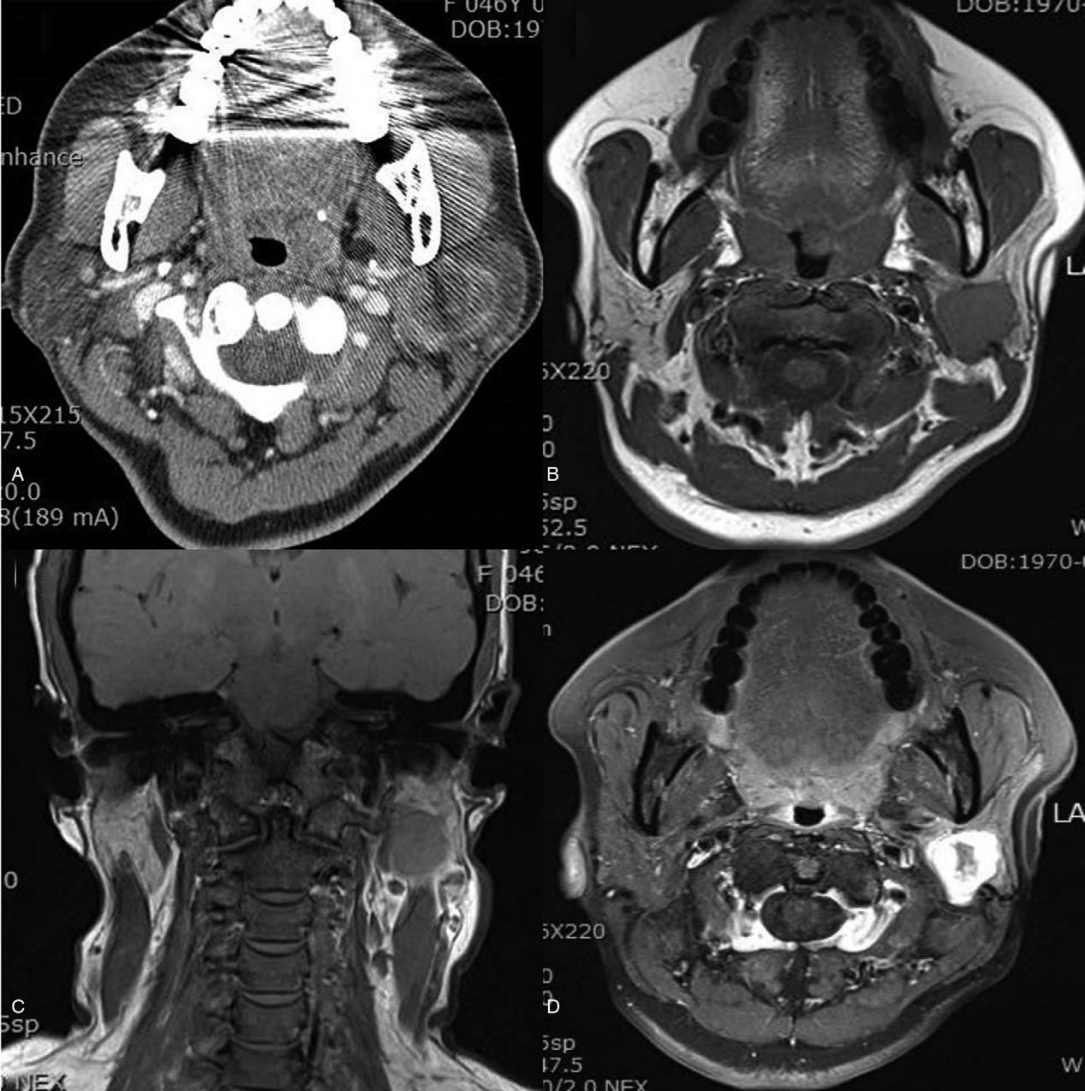
Myxoid liposarcoma with a cyst like appearance at CT and MR imaging. CT image shows hypodence mass in the deep lobe of the parotid ngland (A), Axial (B), and Coronal (C) T1-weighted MR images show a well-defined cystlike lesion in the parotid gland. Axial T2-weighted MR image (D) shows that the mass has heterogeneous high signal intensity.

The patient was a 49-year-old female who presented with a hard fixed mass in the left parotid region. There was no specific past history. Since 3 months before visiting our hospital, the patient complained of a slowly enlarging left parotid mass without facial nerve dysfunction and paralysis. CT images (Fig. 1) revealed a 4 × 3 cm diameter lesion showing low attenuation density and with an irregular thick enhancing wall in the medial portion of her left parotid gland. It was interpreted as a suspicious benign cystic parotid lesion with inflammation or malignancy along with neck node metastasis by the radiologist. This low attenuation lesion had an ill-defined margin and it was localized in the parotid space. There were multiple enlarged lymph nodes at the left cervical chain levels II and III. Further evaluation using MRI showed a mass in the deep lobe of the parotid gland, measuring 3 × 3 cm in size. The mass showed a central hypodense area with close margins to the mandibular ramus and mastoid bone. However, the superficial lobe of the parotid gland was not involved. This mass had a low signal intensity on T1 weighted images and high signal intensity on T2 weighted images (Fig. 1). This lesion was also suspected to be a cystic lesion on MR images. No other abnormalities were noted on a whole body PET-CT imaging scan. Superficial fine-needle aspiration (FNA) was performed on the left parotid mass. Adequate material was obtained for smear and cell block preparations. However, FNA showed no specific features of malignancy.

**Figure 2:**
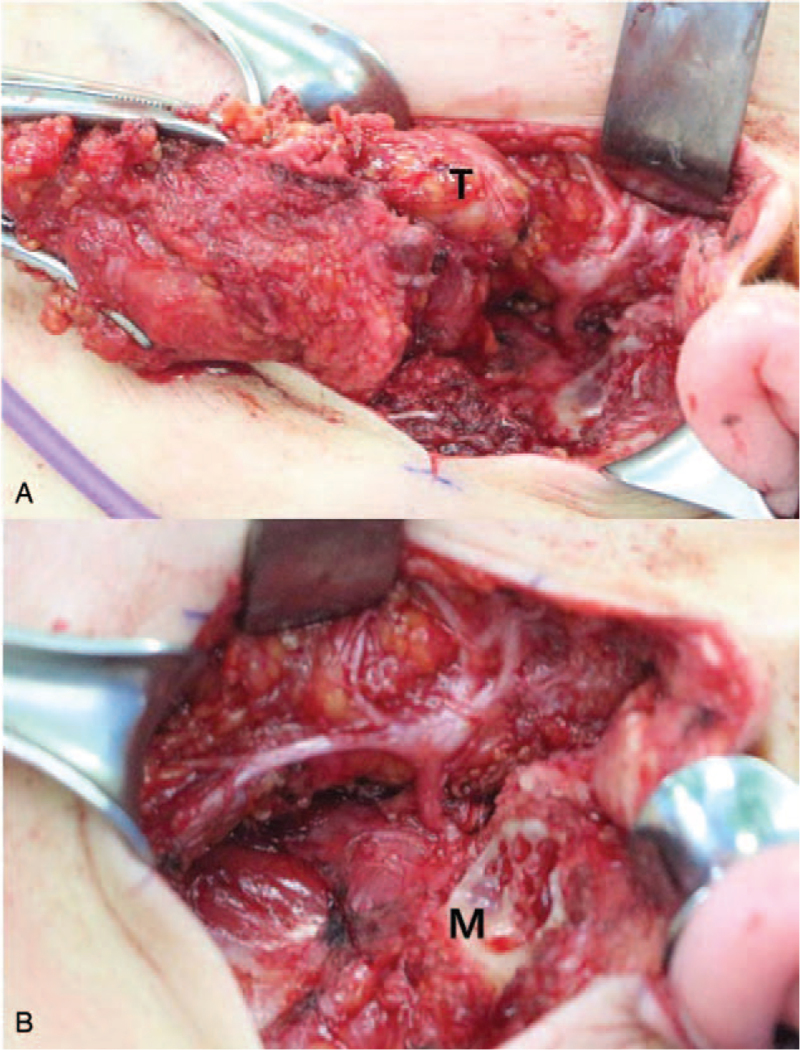
Intraoperative photographies; Tumor (T) was invaded into the deep of the parotid gland (A). For proper identification of facial nerve, mastoidectomy (M) was performed (B).

**Figure 3:**
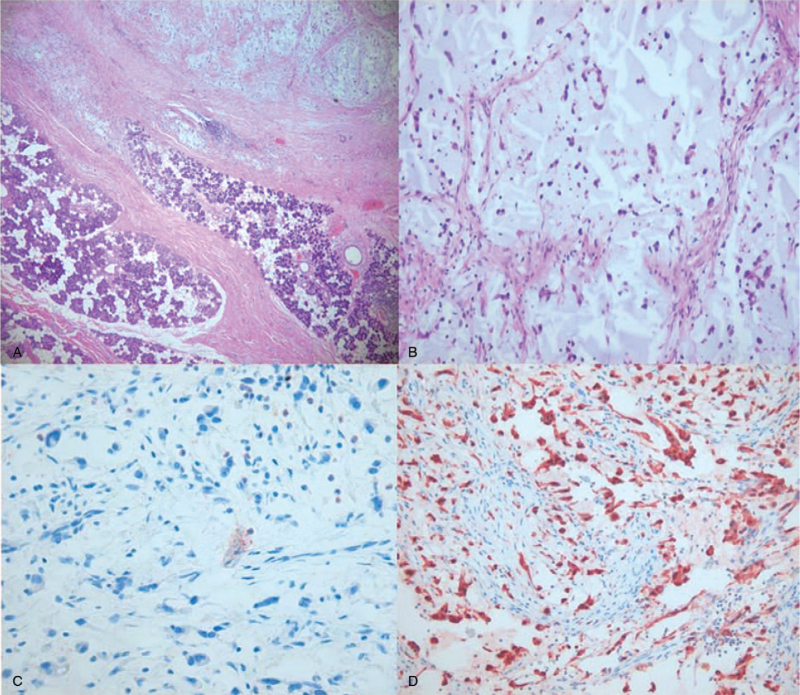
Photomicrographs (A; 40×, B;100×, hematoxylin-eosin stain) shows variable cellular density, dense proliferation of atypical round cells, and mitotic figures. On immunohistochemical stains show CK (C) negative and S-100 (D) diffuse positive.

We performed total parotidectomy after modified radical neck dissection. The mass was deeply located in the deep lobe of the parotid gland and it showed adherence to the surrounding tissues, mandibular ramus, and mastoid bone. While dissecting the parotid gland, it was very difficult to identify the trunk of the facial nerve. The deep portion of the facial nerve was found to be completely engulfed by the tumor. The trunk of the facial nerve was well exposed after removal of the mastoid bone. The mass was successfully dissected from the facial nerve and surrounding tissues (Fig. 2). On immunohistochemical staining, cells were positive for Vimentin and S-100, and they were negative CK, CK7, EMA, and HMB45 (Fig. 3). Therefore, the parotid mass was considered to be a liposarcoma, extraskeletal mesenchymal chondrosarcoma, or malignant peripheral nerve sheath tumor, which may show S-100 positivity on immunohistochemical staining. However, carcinosarcoma was excluded because no distinct carcinomatous component was found in the tumor. A malignant peripheral nerve sheath tumor was also excluded because the shape of the tumor cells made it difficult to visualize the origin of the tumor from the nerve sheath. Finally, this case was diagnosed as primary MLS of the parotid gland. Pathology report showed 2.5 cm sized parotid tumor with lymph node metastasis to 7 out of 31 lymph nodes. Immediate facial nerve function was House Brackmann Grade (HBG) III and recovered within 3 months after the surgery. Other postoperative course was uneventful. The patient received radiation therapy 1 month after surgery. Follow-up period is 57 months and there was no recurrence until now.

## 3 Discussion

The development of liposarcoma is not yet well clarified, but it is thought to arise spontaneously due to genetic mutations. Recent studies do not support the claim that a liposarcoma arises from a lipoma and that there are no specific risk factors. Genetic molecular studies may play a significant role in identifying the tumor type and taking appropriate treatment decisions.^[4,5]^ Chromosomal abnormality such as FUS-DD1T3 causes chromosomal translocation abnormality namely t(12;16)(q13;p11), and chromosomal abnormality such as EWSR1-DD1T3 causes chromosomal translocation abnormality namely t(12;22)(q13;q12).^[12]^

The main signs and symptoms are the presence of a firm and visible swelling in the involved parotid area and a tumor that has been slowly increasing in size. Initially, the overlying skin on the parotid gland may not appear inflamed or show color changes, but changes in the overlying skin including ulceration may be noted as the tumor progresses and becomes larger in size. Also, it may cause neurological signs and symptoms, such as facial weakness and pain, due to facial nerve paralysis.^[4,6,9]^

For making the correct diagnosis of MLS, radiologic images (CT and MRI) are needed.^[10,11]^ In general, lipomas and well-differentiated liposarcomas typically show high signal intensity secondary to the relatively high fat content. MLS generally shows high-signal-intensity foci within a predominantly homogeneous mass on T1-weighted images and intermediate or more prominent signal intensity on T2-weighted images. These foci represent fat within the tumor and usually appear lacy or linear and amorphous rather than solid.^[8]^ However, some MLSs may not exhibit the signal intensity typical of a fatty tumor. They may appear as cystic masses on non-enhanced images. This MLS consisted of a myxoid matrix as the predominant component and small amounts of mature fat within the tumor. Therefore, it showed low signal intensity on nonenhanced T1-weighted images and high signal intensity on T2-weighted images.^[11]^

Because of different morphological lesions associated with liposarcomas, FNAC has certain limitations and it may not be helpful as a diagnostic tool. An open surgical biopsy is more preferred and Hematoxylin and Eosin staining is needed. Special studies such as immunohistochemical stains, molecular testing, and electron microscopic studies are necessary to assist in the diagnosis. Histologically, the tumor reveals variable cellular density, dense proliferation of slightly atypical round cells and rare mitotic figures. Any lesion consisting of edema, an extracellular matrix with a high level of mucopolysaccharide, hyaline cartilage content, and necrosis may appear as a cystic mass.^[4,6]^ For differentiating between carcinoma and sarcoma, special immunohistochemical stains using Vimentin, S-100, CK, CK7, EMA, and HMB45 are needed. In sarcomas, cells are positive for Vimentin and S-100, and they are negative for CK, CK7, EMA, and HMB45. The possibility of extraskeletal mesenchymal chondrosarcoma and malignant peripheral nerve sheath tumor should also be excluded. Carcinosarcoma can be excluded because of no distinct carcinomatous component in the tumor. A malignant peripheral nerve sheath tumor can also be excluded because of the shape of the tumor cells originating from the nerve sheath.

The treatment of choice for MLS of the salivary gland is wide surgical excision with removal of the entire tumor.^[9]^ Following surgery, radiation therapy is usually provided for tumors in the head and neck region. When the tumor is situated at an inaccessible location such as base of the skull or the parapharyngeal space, chemotherapy, and radiation therapy may be considered as noninvasive procedures. Careful follow-up with regular work-up is very important to detect recurrence and any metastatic lesions. Data on the prognosis of MLS of the salivary gland is not available due to the rarity of the cancer. In general, the prognosis of MLS of the salivary gland is generally good after prompt diagnosis and treatment. However, it shows poor prognosis in the advanced stage, high cell division rate, and large tumor size.^[4]^

In conclusion, we report an unusual case of primary MLS of the parotid gland. Radiologically, some MLSs may not exhibit the signal intensity typical of a fatty tumor and may instead appear as cystic masses. The absence of high-signal-intensity foci on T1-weighted images makes an MLS indistinguishable from other soft-tissue masses. Therefore, MLS should be differentiated from benign cystic lesions and other malignant lesions using immunohistochemical staining and MR images.

## Author contributions

**Conceptualization:** Yong Tae Hong.

**Formal analysis:** Yunna Yang.

**Investigation:** Yunna Yang.

**Methodology:** Yong Tae Hong.

**Resources:** Hyunjun Lee.

**Validation:** Hyunjun Lee.

**Visualization:** Hyunjun Lee.

**Writing – original draft:** Yong Tae Hong.

**Writing – review & editing:** Yong Tae Hong.
